# A method to identify differential expression profiles of time-course gene data with Fourier transformation

**DOI:** 10.1186/1471-2105-14-310

**Published:** 2013-10-18

**Authors:** Jaehee Kim, Robert Todd Ogden, Haseong Kim

**Affiliations:** 1 Department of Statistics, Duksung Women’s University, Seoul, South Korea; 2 Department of Biostatistics, Columbia University, New York, USA; 3 Biochemicals and Synthetic Biology Research Center, Korea Research Institute of Bioscience & Biotechnology, Daejeon, South Korea

## Abstract

**Background:**

Time course gene expression experiments are an increasingly popular method for exploring biological processes. Temporal gene expression profiles provide an important characterization of gene function, as biological systems are both developmental and dynamic. With such data it is possible to study gene expression changes over time and thereby to detect differential genes. Much of the early work on analyzing time series expression data relied on methods developed originally for static data and thus there is a need for improved methodology. Since time series expression is a temporal process, its unique features such as autocorrelation between successive points should be incorporated into the analysis.

**Results:**

This work aims to identify genes that show different gene expression profiles across time. We propose a statistical procedure to discover gene groups with similar profiles using a nonparametric representation that accounts for the autocorrelation in the data. In particular, we first represent each profile in terms of a Fourier basis, and then we screen out genes that are not differentially expressed based on the Fourier coefficients. Finally, we cluster the remaining gene profiles using a model-based approach in the Fourier domain. We evaluate the screening results in terms of sensitivity, specificity, FDR and FNR, compare with the Gaussian process regression screening in a simulation study and illustrate the results by application to yeast cell-cycle microarray expression data with alpha-factor synchronization.

The key elements of the proposed methodology: (i) representation of gene profiles in the Fourier domain; (ii) automatic screening of genes based on the Fourier coefficients and taking into account autocorrelation in the data, while controlling the false discovery rate (FDR); (iii) model-based clustering of the remaining gene profiles.

**Conclusions:**

Using this method, we identified a set of cell-cycle-regulated time-course yeast genes. The proposed method is general and can be potentially used to identify genes which have the same patterns or biological processes, and help facing the present and forthcoming challenges of data analysis in functional genomics.

## Background

Time-course gene expression data are often measured to study dynamic biological systems and gene regulatory networks. Array technologies have made it straightforward to monitor the expression pattern of thousands of genes simultaneously. The challenge now is to interpret such massive data sets. The first step is to extract the fundamental patterns of gene expression inherent in the data. Gene-expression levels can be monitored with cDNA or oligonucleotide chips over a time-course for a temporal process. Following a microarray time series experiment, a key challenge is to extract the continuous representation of all genes throughout the time-course. Identifying significant or differentially expressed genes is challenging because different genes may have different profiles, and because of the noise present in time series expression data. A comprehensive review about time series expression data analysis and the related computational challenges may be found in [1].

Microarrays have recently been used for the purpose of monitoring expression levels of thousands of genes simultaneously and for identifying genes that are differentially expressed. With the number of inferences made in the analysis of microarray data, it is natural to be concerned about multiple testing. This problem of multiplicity can be dealt with by controlling the false discovery rate (FDR) [2].

In the past decade, many approaches to gene selection have been considered including a two sample t-test [3], a regression approach [4], and a mixture model approach [5]. Other approaches to this problem include the Empirical Bayesian (EB) method [6] and the Significance Analysis of Microarray (SAM) method [7]. The multiplicity problem is addressed in adopting a resampling-based approach to controlling FDR [8]. Also an ANOVA formulation and an empirical Bayes adjustment to the t-statistics [9] and an empirical Bayes screening procedure have been proposed [10].

There has been considerable research about discovering patterns using clustering and testing including clustering after transformation and smoothing as a technique for nonparametrically estimating and clustering a large number of curves [11] and clustering short time series gene expression data by selecting a set of potential expression profiles [12].

Smoothing away noise-induced wiggles of gene expression data with Fourier series for microarray data has been considered including an improved Fourier method with irregular or monotonic components of cell-cycle expression [13], a two-step procedure for clustering periodic patterns of gene expression profiles using a Fourier series approximation with frequency and amplitude of order one [14], a multivariate modeling approach using partial least squares (PLS) regression to identify genes with periodic fluctuations in the budding yeast cell cycle data [15], a Hidden Markov Models (HMMs) approach to account for the horizontal dependencies along the time axis [16], and a model-based clustering of the Fourier coefficients calculated on the first difference of the time-course data [17].

Model-based hierarchical clustering was proposed in character recognition problems using a multivariate normal model [18] and it may be used to guide the choice of the model based on computing an approximate maximum for the classification likelihood [19].

There has been much work done on clustering microarray data, mostly on grouping common expression patterns. However, less attention has been paid to time-course gene studies. Currently the analysis of GETS (gene expression time-series) is commonly performed using a GP (Gaussian process) [20-24]. Also a Bayesian analysis of microarray time series has been developed with the software package BATS [25].

In this research, we propose a new method for gene screening using Fourier coefficients to cluster time-courses of genes that exhibit similar patterns.

This paper introduces a methodology for gene selection based on time-course data. The first step is screening, in which we seek to isolate the inactive genes from the active ones, while properly taking into account the serial dependence in the time course data and controlling the FDR, all in the Fourier domain. The second step involves a model-based clustering of the “active” genes, also in the Fourier domain. We evaluate the performance of the methodology using both simulated data and yeast cell cycle data.

## Results and discussion

### Simulated data

Since real expression data sets are generally noisy and their clusters may not be fully reflective of the class information, we first evaluate the performance of our method with simulated data, for which the “true” classes are known.

We simulate data according to the regression model

$$Y_{\text{iu}}=f_{i}\left(t_{\text{iu}}\right)+\epsilon_{\text{iu}}\;i=1,2,\cdot \cdot \cdot n,u=1,2,\cdot \cdot \cdot ,m$$

where *n* = 800 genes, *m* = 20 time points, and *E*(*ϵ*_*iu*_) = 0 and *ϵ*_*iu*_’s from an autoregressive AR(1) process with a variety of values of the AR parameter. The regression functions for *f* are:

$$f_{1}\left(t\right)=0$$

$$f_{2}\left(t\right)=10t$$

$$f_{3}\left(t\right)=\min\left\{\left(\frac{2-5t}{2}\right),\left({\left(\frac{5t-2}{3}\right)}^{2}+\sin\frac{5\pi t}{2}\right)\right\}$$

$$f_{4}\left(t\right)=-f_{3}\left(t\right)$$

$$f_{5}\left(t\right)=2\cos\left(2\pi t\right)$$

$$f_{6}\left(t\right)=5+f_{5}\left(t\right)$$

Each simulated dataset consists of 800 curves originating from the 6 functions: 400 *f*_1_, s and 80 curves of each *f*_2_, ⋯, *f*_6_, to reflect typical gene expression data. Thus, there are 5 sets of differential genes and 1 set of non-differential genes. The standard deviation of the innovation process was set to *σ* = 0.5 and *σ* = 1.5 to represent low and high noise situations, respectively.

The cosine system $\left\{\sqrt{2}\cos(\pi \text{jt})\right\}$ is used as the set of basis functions. Though the optimal choice for *J* could vary from function to function, we choose to use a single smoothing parameter that performs reasonably well for all of the curves. In the simulation ten Fourier coefficients are used for the spectrum estimation. Several numbers of Fourier coefficients are considered for test statistics in the proposed screening method.

The number of clusters is determined according to the Bayesian Information Criterion (BIC).

Let *T* be a clustering map defined as

$$T\left(f,g\right)=\left\{\begin{array}{c} 1,\\ 0, \end{array}\;\right)\begin{array}{c} \begin{array}{cc} \text{if}\;f\;\text{and}\;g\;\text{are}\;\text{in}\;\text{the}\;\text{same}\;\text{cluster} & \end{array}\\ \begin{array}{cc} \begin{array}{ccccc} \begin{array}{ccc} \begin{array}{cc} \text{otherwise}. & \end{array} & & \end{array} & & & & \end{array} & \end{array} \end{array}$$

Regarding the estimation error, the clustering estimation error rate *η*(*K*) depending on *K* clusters is defined as

$$\eta \left(K\right)=\frac{2}{n\left(n-1\right)}\sum_{r<s}I(T_{K}(f_{r},f_{s})\neq {\hat{T}}_{K}({\hat{f}}_{r},{\hat{f}}_{s}))$$

where {*f*_1_, ⋯, *f*_*n*_} denote the true curves and $\left\{{\hat{f}}_{1},\cdots ,{\hat{f}}_{n}\right\}$ denote the estimated curves. Let *T* and $\hat{T}$ represent the corresponding cluster maps, and *K* denote the number of clusters. *n(K)* is the fraction of all pairs that are incorrectly placed in different clusters.

We compare our screening method with the recently proposed GPR (Gaussian process regression) screening [24]. GPR seeks to quantify the true signal and noise in a gene expression time series, allows us to rank the differential expression of the gene profile. A Gaussian process using a squared-exponential covariance function is based on the assumption that the underlying true signal in a profile is a smooth function. GPR is applied to each gene curve with the assumption that each gene can be categorized as either quiet or differentially expressed. The genes may then be ranked in decreasing order according to their values of likelihood ratio (LR) test statistics for testing between these two characterizations. The optimal critical value for the LR test statistics for GPR screening was empirically determined to be log(1.5) based on the true positive rate (sensitivity) since in the simulation, this cutoff allowed more than 80% of differentially expressed genes to be detected. There is no clear-cut critical value for this scheme. The genes greater than the critical value are detected as differential. After the differential genes are obtained with GPR screening, clustering procedure can be done with those genes.

To summarize the performance across the 500 replications of 800 curves in each data set, we compute four performance measures to evaluate the procedures:

i. Sensitivity: proportion of differentially expressed genes that are declared significant

ii. Specificity: proportion of non-differentially expressed genes that are not declared significant

iii. False discovery rate (FDR): proportion of genes declared significant that are not differentially expressed

iv. False non-discovery rate (FNR): proportion of genes not declared significant that are differentially expressed

Tables 1 and 2 show the clustering estimation error rates, average silhouette values and Adjusted Rand Index values (See Section on ‘Performance Metrics’) for the model-based clustering without screening versus with the proposed screening according to the positive correlation. They also show the sensitivity, specificity, FDR and FNR of our proposed screening procedure and GPR screening.

**Table 1:** Comparison of screening and clustering results (low noise)

			**Without screening**			**With screening**						
AR(1) parameter	Method	*J*	Error	Sil	ARI	Error	Sil	ARI	Sensitivity	Specificity	FDR	FNR
*p = 0.1*	FC*	2	.037	.509	.909	.015	.560	.918	.878	.723	.121	.276
		3	.020	.471	.921	.016	.484	.932	.860	.783	.139	.216
		4	.015	.430	.963	.017	.438	.937	.863	.842	.136	.157
		5	.015	.388	.964	.014	.403	.944	.854	.798	.145	.201
		8	.015	.305	.964	.017	.317	.940	.851	.836	.148	.163
	GPR**								.855	.779	.220	.144
*p = 0.2*	FC	2	.052	.471	.871	.021	.523	.875	.871	.722	.128	.277
		3	.036	.423	.912	.026	.443	.888	.846	.783	.153	.217
		4	.029	.386	.931	.028	.398	.895	.847	.839	.152	.160
		5	.027	.348	.935	.022	.366	.906	.837	.798	.162	.205
		8	.028	.274	.936	.029	.287	.895	.830	.836	.169	.163
	GPR								.826	678	.321	.173
*p = 0.3*	FC	2	.073	.430	.822	.030	.487	.815	.863	.723	.136	.276
		3	.056	.380	.865	.042	.402	.814	.828	.783	.171	.217
		4	.052	.339	.875	.045	.356	.825	.827	.834	.172	.165
		5	.049	.306	.883	.036	.326	.845	.817	.790	.182	.209
		8	.047	.244	.888	.049	.257	.823	.803	.832	.196	.167
	GPR								.798	.571	.428	.201
*p = 0.5*	FC	2	.159	.340	.610	.056	.414	.633	.835	.717	.165	.201
		3	.139	.287	.663	.093	.329	.591	.775	.768	.224	.231
		4	.124	.255	.702	.113	.280	.578	.766	.811	.233	.188
		5	.132	.226	.682	.093	.259	.615	.762	.773	.237	.226
		8	.143	.181	.649	.134	.205	.562	.730	.815	.269	.184
	GPR								.756	.410	.589	.244
*p = 0.7*	FC	2	.266	.287	.345	.088	.357	.351	.755	.704	.244	.295
		3	.264	.224	.347	.153	.272	.314	.682	.738	.317	.261
		4	.258	.190	.370	.186	.230	.303	.668	.771	.331	.228
		5	.258	.171	.375	.161	.211	.317	.676	.745	.324	.255
		8	.267	.137	.339	.220	.172	.287	.641	.769	.358	.230
	GPR								.731	.335	.664	.268

* FC: proposed method with Fourier coefficients, **GPR: Gaussian process regression.

Comparison of estimation error rate (E), Silhouette width (S) and Adjusted Rand Index (ARI) values of model-based clustering without screening vs with screening with *J* Fourier coefficients including sensitivity, specificity, FDR and FNR with m = 20 time points. These summaries are based on 500 repetitions of each consisting of 800 curves with AR(1) parameter *ρ*’s with the noise standard deviation *σ* = 0.5.

**Table 2:** Comparison of screening and clustering results (high noise)

			**Without screening**			**With screening**						
AR(1) parameter	Method	*J*	Error	Sil	ARI	Error	Sil	ARI	Sensitivity	Specificity	FDR	FNR
*p = 0.1*	FC*	2	.326	.259	.200	.235	.292	.149	.589	.708	.411	.291
		3	.321	.192	.199	.298	.214	.151	.561	.716	.439	.283
		4	.321	.151	.194	.320	.166	.156	.553	.722	.447	.278
		5	.323	.125	.185	.269	.142	.156	.571	.714	.428	.285
		8	.324	.084	.175	.324	.094	.148	.552	.722	.448	.277
	GPR**								.483	.779	.221	.517
*p = 0.2*	FC	2	.343	.253	.164	.234	.291	.117	.567	.697	.432	.302
		3	.339	.185	.155	.287	.208	.117	.545	.703	.454	.296
		4	.338	.149	.151	.306	.160	.120	.539	.708	.461	.291
		5	.337	.125	.146	.261	.138	.120	.555	.702	.445	.297
		8	.338	.086	.132	.307	.094	.113	.538	.706	.462	.293
	GPR								.536	.677	.323	.463
*p = 0.3*	FC	2	.359	.248	.128	.284	.290	.090	.546	.681	.453	.318
		3	.351	.185	.119	.329	.208	.089	.531	.683	.468	.316
		4	.350	.148	.115	.347	.159	.092	.526	.685	.473	.314
		5	.350	.127	.108	.304	.137	.088	.537	.681	.462	.318
		8	.357	.083	.089	.351	.091	.079	.526	.685	.474	.314
	GPR								.584	.572	.427	.415
*p = 0.5*	FC	2	.383	.246	.073	.330	.284	.053	.517	.632	.482	.367
		3	.375	.183	.066	.356	.198	.051	.512	.634	.488	.365
		4	.369	.151	.062	.365	.158	.052	.510	.633	.490	.366
		5	.369	.126	.056	.338	.137	.046	.514	.633	.485	.367
		8	.370	.086	.046	.370	.092	.042	.509	.634	.490	.365
	GPR								.646	.409	.590	.353
*p = 0.7*	FC	2	.395	.248	.035	.356	.275	.030	.505	.504	.495	.422
		3	.384	.186	.034	.368	.193	.028	.503	.503	.496	.424
		4	.383	.148	.031	.373	.155	.026	.502	.502	.497	.421
		5	.381	.125	.028	.358	.134	.024	.504	.504	.496	.419
		8	.377	.092	.027	.370	.097	.023	.503	.502	.497	.415
	GPR								.679	.337	.662	.320

* FC: proposed method with Fourier coefficients, **GPR: Gaussian process regression

Comparison of estimation error rate (E), Silhouette width (S) and Adjusted Rand Index (ARI) values of model-based clustering without screening vs with screening with *J* Fourier coefficients including sensitivity, specificity, FDR and FNR with m = 20 time points. These summaries are based on 500 repetitions of each consisting of 800 curves with AR(1) parameter ρ s with the noise standard deviation *σ* = 1.5.

As shown in Table 2 the error rates of the high noise case are bigger and the sensitivity is smaller than those of the lower noise case in Table 1. However the high noise level does not diminish the performance of the screening procedure. From Tables 1 and 2 it can be seen that the clustering estimation error is smaller after screening than it is without screening. Also, the clustering estimation error becomes smaller as the number of Fourier coefficients *J* becomes larger and for smaller values of the AR parameter. Table 2 (higher noise level) shows that the screening percentage of our method is a little higher than GPR for some choices of *J*. Overall, the proposed method demonstrates improved sensitivity according to the number of Fourier coefficients *J* and much improved specificity and FDR as compared to GPR. In addition, the proposed method has an advantage that it does not require estimation of the covariance structure

### Yeast cell cycle data analysis

We have used alpha synchronized yeast cell expression data [26] available at http://genome-www.stanford.edu/cellcycle/ to test our algorithm. After removing genes with missing values, there were 4,489 genes remaining out of 6178 genes. This data contained 18 time points sampled uniformly every 7 min between 0 and 119 min.

Following [27], we assumed a first-order auto-correlation structure for the error terms. The method was repeated for each of several choices for *J*. Table 3 shows the median and the average silhouette values with Euclidean distance between samples by model-based clusterings for various *J* values both with and without the screening step using a significance level of FDR 5%. The number of genes with differentially expression is 2,227 out of 4,489 at the significance level *α = 0.05*.

**Table 3:** Silhouette values for model-based clustering with Fourier coefficients of yeast data

**Number. of Fourier coeff**	**Number of clusters without-screening**	**Number of clusters with screening**	**No. of sig. genes**	**Without-screening**		**Screening**	
				**Med sil**	**Avg. sil**	**Med sil**	**Avg. sil**
J = 2	5	4	1715	.160	.112	.451	.388
J = 3	5	4	1735	.204	.146	.505	.426
J = 4	5	4	2227	.174	.119	.552	.485
J = 5	5	4	2792	.029	.015	.048	.028
J = 6	5	4	3071	.196	.119	.041	.043
J = 8	5	4	3050	.136	.055	.032	.024
J = 10	5	4	3142	.153	.092	.031	.010

Median and average silhouette values for model-based clustering with Fourier coefficients using Euclidean distance with screening vs. without screening.

**Figure 1:**
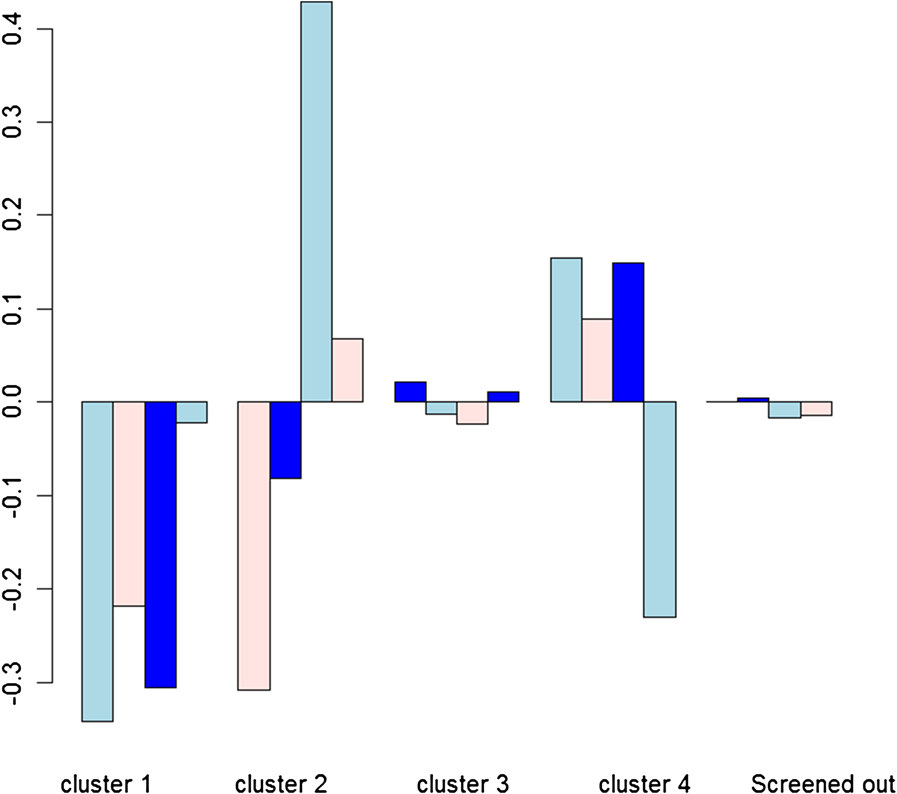
**Means of J = 4 sample Fourier coefficients with yeast data.** The mean profiles of Fourier coefficients in the four clusters and one cluster with genes screened out.

**Figure 2:**
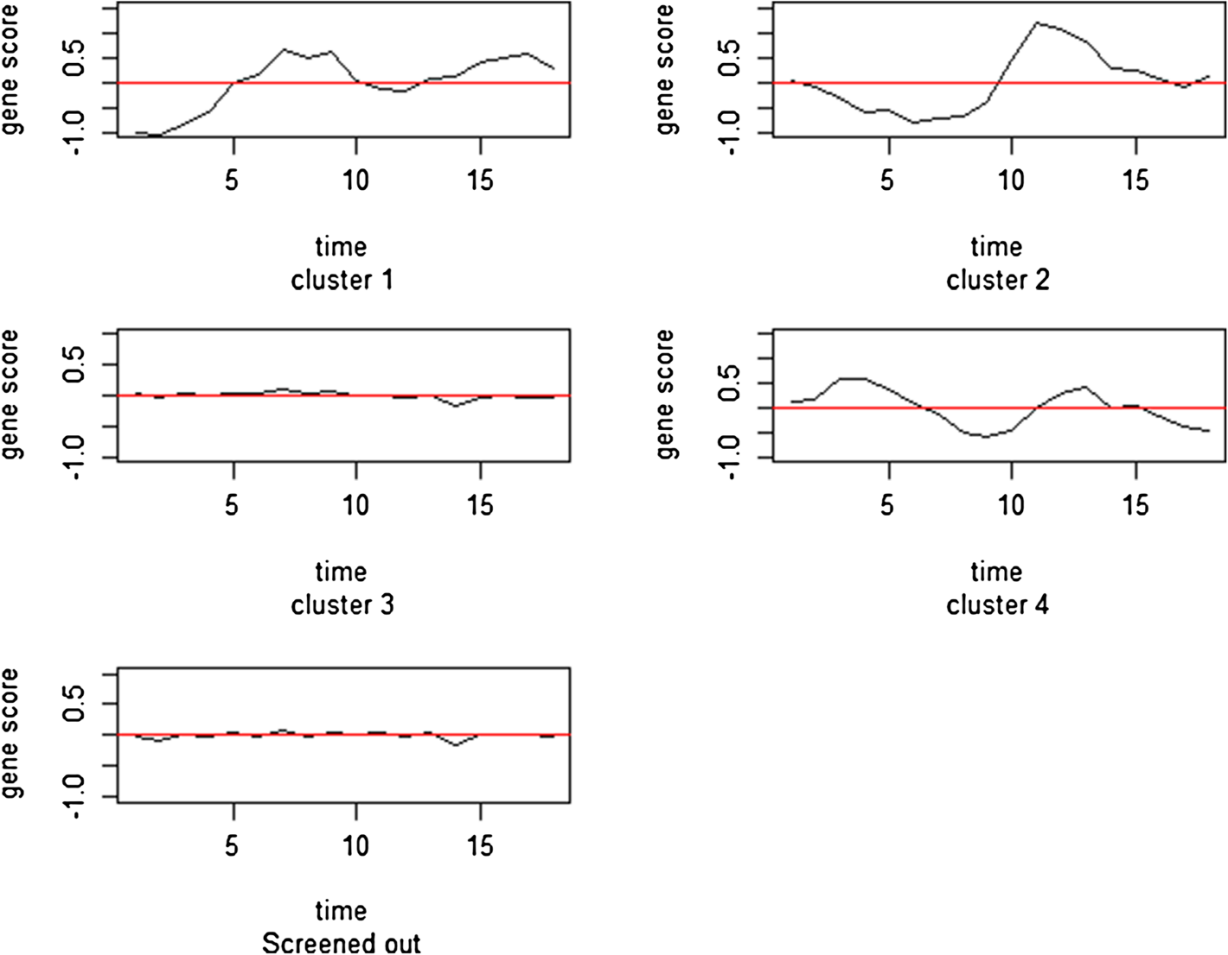
**Average gene curves in four clusters and one screened-out cluster.** The mean curves of gene curves in 4 clusters and one mean curve with genes screened out.

Judging from the silhouette value, the model-based with 4 Fourier coefficients and 4 clusters was considered most appropriate. Therefore it should be noted that silhouette values of Euclidean distance between two clustering models may not be the only criterion for model comparison. Rather, as in the following gene ontology analysis, clustering should be evaluated based on biological interpretation of results. With *J = 4*, the model-based clustering approach results in 4 clusters consisting of 51, 29, 2077, and 70 genes, respectively. Figure 1 shows plots of the sample Fourier coefficients. Figure 2 shows pointwise means of Fourier estimated gene scores in each cluster with *J = 4* sample Fourier coefficients. The graph in the bottom left-hand corner of Figure 2 shows the estimated mean of gene scores of screened out. Gaussian mixture model clustering allows clusters to have different orientation or sizes while preserving some common features, such as an ellipsoidal shape. Clusters 1, 2 and 4 in particular show a cycle while cluster 3 consists of less active genes.

We also applied GPR screening for yeast data with the critical value log (1.5) and identified 1,620 non-quiet differentially expressed genes. A Gaussian mixture model clustering algorithm was applied to these genes indicating 4 clusters. The clusters have 209, 477, 598, and 336 genes, respectively. For this clustering the median silhouette value is 0.508 and the average silhouette value is 0.370, each less than the corresponding silhouette summaries for the proposed procedure.

Owing to noise and the high dimensionality of data, careful consideration of statistical and biological validity is needed when analyzing microarray data. From our review we have found that without plausible interpretation and biological validation, the number of partitions produced by numerical analysis is highly unreliable, and sometimes even misleading.

In order to evaluate our clustering analysis, Gene Ontology (GO) is applied to the clustered genes [28]. GO consists of three organizing principles: biological processes, molecular functions, and cellular components. Biological process evidences will be used in this study because the yeast dataset was obtained from the cell cycle process. The GO database provides useful tools to annotate and analyze a set of genes. For example, GOstat searches for statistically overrepresented GO annotations by evaluating the significance of functional processes and molecular mechanisms [29]. This tool simply derives the statistical significance between expected and observed functional categories based on the Fisher’s exact test. So we firstly apply this tool to our four clusters and select significantly overrepresented GO terms in each cluster with a criterion p-value < 0.001. Along with the four clusters, we also look for the significant GO terms of the screened 2,227 genes for the comparison. Before performing the hypergeometric tests, we filtered out the genes which cannot be identified from the Yeast annotation database (R package: org.Sc.sgd.db). Table 4 shows the number of genes in each cluster before and after the filtering and the selected (overrepresented) GO terms from the hypergeometric test of the clustered genes.

**Table 4:** Number of genes in each cluster with J = 4

	**Screened out**	**Cluster 1**	**Cluster 2**	**Cluster 3**	**Cluster 4**
Number of genes	2262	51	29	2077	70
Filtered genes	2041	46	28	1881	64
Significant GO terms	1	36	17	6	37

The number of genes screened, screened out and significant genes with respect to GO.

Even though the number of genes affects to the hypergeometric test, cluster 3 and the screened-out group have only one and six significantly overrepresented GO terms, respectively. This means that all the genes (or some of them) in those clusters rarely share their biological processes and these clusters can be considered as random collections of genes. So, we focused on the three clusters 1, 2, and 4 to find their biological meanings.

**Figure 3:**
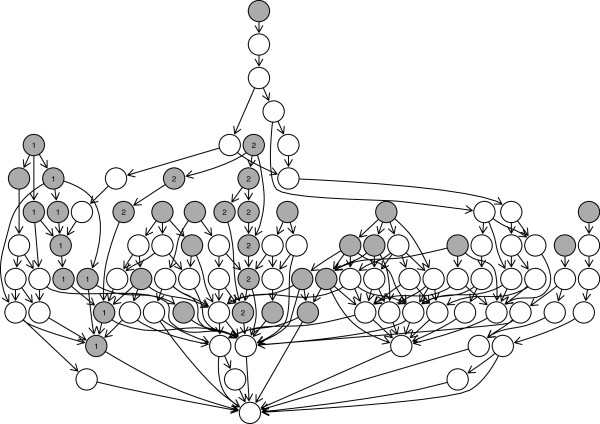
GO graph of each cluster of yeast data.

In order to identify the relationships among the selected GO terms, we construct a GO graph of each cluster. This graph is constructed by locating the selected terms as leaf nodes and linking all the nodes to their ancestors until their root term. Note that whatever GO terms are selected in a cluster, the cluster has only one GO relationship graph because every GO terms are eventually ended up at the root term if we follow their ancestors. Figure 3 depicts the GO graphs of the three clusters. Gray colored nodes represent selected GO terms in each cluster. We realize that most of the selected GO terms are related with each other as a parent–child relationship and they tend to be closely located in each graph.

Among the selected GO terms, we looked into the terms which are connected sequentially from the top node to the bottom (root). This cascade form of GO terms provides more enhanced evidences that the corresponding genes are more closely related in their biological processes than that of a parent–child relationship. These sequentially connected terms are labeled by their subset number in the Figure 3 and are summarized in Table 5. In cluster 1, subset 2 includes the genes mainly affect to cell cycle especially Mitosis. Mitosis is a division process that produces two daughter cells having the same genetic information as their parent cell. During this Mitosis, structural assembly of DNA and nucleosome is processed, which can be represented in the subset 1 of cluster 1. Mitosis is followed by cytokinesis and the cell wall of the daughter cells is formed, whose related genes are shown in cluster 2. In cluster 4, genes in subset 3 are in charge of DNA replication in the S phase of cell cycle. So, our method enables us to identify the genes that are expressed in dynamical cellular events of DNA duplication and cell division. Though the subset 1 and 2 of cluster 4 seem to be more related with the responses to DNA stresses then cell cycle, it is known that heat shock proteins are also responsible for cell proliferation and cell cycle ([30,31]). Therefore, based on this result, further experimental investigation could be possible to reveal the link between two different biological processes, “heat-shock response” and “cell cycle”.

**Table 5:** GO terms connected sequentially in their GO relationship graph (C: cluster number, S: subset number)

**C**	**S**	**GO ID**	**Terms**	**S**	**GO ID**	**Terms**
1	1	GO:0006334	Nucleosome assembly	2	GO:0000280	Nuclear division
		GO:0031497	Chromatin assembly		GO:0000087	M phase of mitotic cell cycle
		GO:0006323	DNA packaging		GO:0048285	Organelle fission
		GO:0034728	Nucleosome organization		GO:0022402	Cell cycle process
		GO:0006333	Chromatin assembly or disassembly		GO:0000278	Mitotic cell cycle
		GO:0016043	Cellular component organization		GO:0007049	Cell cycle
		GO:0051276	Chromosome organization		GO:0007067	Mitosis
		GO:0006325	Chromatin organization		GO:0000279	M phase
		GO:0006996	Crganelle organization		GO:0022403	Cell cycle phase
2	1	GO:0007109	Cytokinesis, completion of separation	2	GO:0071554	Cell wall organization or biogenesis
		GO:0000920	Cell separation during cytokinesis		GO:0007047	Cellular cell wall organization
		GO:0032506	Cytokinetic process		GO:0071555	Cell wall organization
		GO:0000910	Cytokinesis		GO:0070882	Cellular cell wall organization or biogenesis
					GO:0031505	Cungal-type cell wall organization
4	1	GO:0051716	Cellular response to stimulus	2	GO:0006302	Couble-strand break repair
		GO:0050896	Response to stimulus		GO:0006281	DNA repair
		GO:0033554	Cellular response to stress		GO:0000724	Couble-strand break repair via homologous recombination
		GO:0006950	Response to stress		GO:0006974	Cesponse to DNA damage stimulus
		GO:0034605	Cellular response to heat	3	GO:0006260	DNA replication
		GO:0009408	Response to heat		GO:0006273	Cagging strand elongation
		GO:0009628	Response to abiotic stimulus		GO:0006261	DNA-dependent DNA replication
		GO:0009266	Response to temperature stimulus		GO:0006271	DNA strand elongation during DNA replication
					GO:0022616	DNA strand elongation
					GO:0006259	DNA metabolic process

Cluster 1, 2 and 4 have biologically meaningful genes as shown in the table.

## Conclusions

A number of recent studies in this field have focused on the analysis of time series of gene expression data, collected by performing DNA microarray experiments at several or more points in time. We have proposed a significance method to identify differentially expressed genes in time course microarray gene expression data using time series screening based on Fourier coefficients controlling FDR and model based clustering with the sample genewise Fourier coefficients, and have compared our screening method with GP screening. Recently spectral mixture kernels [32] have been introduced with a Gaussian mixture as a Fourier transform of kernels and these kernels are able to discover patterns and extrapolate and model negative covariances, illustrating the relationship between the GP and the Fourier approach.

We demonstrated the effectiveness of our approach using model-based clustering of gene profiles. Although we assumed that the residuals follow an AR process, we found that it is not necessary to assume a specific correlation structure since the sample Fourier coefficient estimates themselves do not depend heavily on the underlying covariance structure. The most commonly used techniques are clustering (unsupervised) techniques, which are particularly well suited for an exploratory investigation of this kind of data. The main advantage of the model-based methods is their reliance on a highly structured theoretical basis. Model-based clustering methods are based on the assumption that the data were generated by some underlying model and attempt to infer these models from data. Data generated by the same model is then considered to be “similar” and clustered together. Also, the choice of the optimal number of clusters and the selection of the best model can be performed using sound statistical criteria.

The proposed method is able to identify a set of cell-cycle-regulated genes in yeast and time-course genes. The proposed method is general and can be potentially used to identify genes which have the same patterns or biological processes, and help facing the present and forthcoming challenges of data analysis in functional genomics.

## Methods

### The Fourier representation model

We observe data *Y*_*iu*_, *u*th observation on the *i*th curve, of the form

(1) $$\begin{array}{ll} Y_{\text{iu}}=f_{i}\left(t_{\text{iu}}\right)+\epsilon_{\text{iu}}\; & \;i=1,2,\cdots n,\\ & u=1,2,\cdots ,m \end{array}$$

where *E*(*ϵ*_*iu*_) = 0 and the *ϵ*_*iu*_ values arise from a covariance-stationary process with mean zero and covariance function *γ*_*i*_, *γ*_*i*_(*k*) = *E*(*ϵ*_*iu*_*ϵ*_*i*,*u* + *k*_) for all *u* and *k*. In a microarray experiment *Y*_*iu*_ is the log gene expression of gene *i* at time *u*. We assume that the data from one curve are independent from those of other curves.

We assume further that the curve *f*_*i*_ belongs to a class of smooth functions as defined below:

(2) $$f_{i}\left(t\right)=\varphi_{i0}+\sum_{j=1}^{\infty }\varphi_{\text{ij}}b_{j}\left(t\right)$$

where {*b*_*j*_} is an orthonormal basis system and

(3) $$\varphi_{\text{ij}}=\int f_{i}\left(t\right)b_{j}\left(t\right)\text{dt}$$

We can estimate each *f*_*i*_ using its empirical Fourier coefficients:

(4) $${\hat{f}}_{i}\left(t\right)={\hat{\varphi }}_{i0}+\sum_{j=1}^{J}{\hat{\varphi }}_{\text{ij}}b_{j}\left(t\right)$$

which is the projection onto the first *J* basis functions where *J* , 1 ≤ *J* ≤ *m*, is a smoothing parameter to be chosen based on the data.

The empirical Fourier coefficients can be computed as

(5) $${\hat{\varphi }}_{i0}=\frac{1}{m}\sum_{r=1}^{m}Y_{\text{ir}}\;\text{and}\;{\hat{\varphi }}_{\text{ij}}=\frac{1}{m}\sum_{r=1}^{m}Y_{\text{ir}}b_{j}\left(t_{r}\right)$$

with *t*_*r*_ *= r/m*. To estimate the true Fourier coefficients, the covariance structure is not considered since the covariance matrix of a finite set of estimated Fourier coefficients is asymptotically proportional to the identity matrix.

### Screening out flat genes

Many microarray experiments are aimed at finding ‘active’ genes that vary significantly in expression. Differential expression indicates the changing of transcription levels across different time points, and it is thought that these transcription changes might be responsible for the change in phenotype. For example, the genes responsible for the presence of a certain disease will be transcribed at a different rate than when the disease is absent. Cluster analysis often fails to detect differentially expressed genes that belong to clusters for which most genes do not change because most of the other genes in their clusters do not change significantly.

The problem can be formulated as hypothesis-testing for individual genes as follows:

*H*_0_ : *f*_*i*_(·) = *C* versus *H*_1_ : *f*_*i*_(·) ≠ *C* for *i* = 1, 2, ⋯ *n*,i.e., for *n* genes, we are considering *n* pairs of mutually exclusive hypotheses:

*H*_*0*_: Gene *i* is not differentially expressed.

*H*_*1*_: Gene *i* is differentially expressed.

In a microarray setting, it is typical to consider thousands of tests simultaneously. In this situation the familywise error rate (FWER) or FDR (false discovery rate), the average proportion of inactive genes among those that were declared active, should be controlled. The FDR procedure [2] is as follows:

Let *k* be the largest *g*, 0 ≤ *g* ≤ *n*, for which

$$P_{\left(g\right)}\leq \frac{g\alpha }{n}$$

Then reject all *H*_(*g*)0_, for *g* = 1, 2, ⋯, *k*, where *H*_(*g*)0_ is the associated null hypothesis and *P*_(*g*)_ is the *g*th smallest p-value among all the p-values calculated for each of the hypotheses. For all genes, we apply a first-order auto-regressive (AR(1)) process to model the time dependency of the data. For testing change in the mean function of time series data, the test [32] rejects the null hypothesis of no change for large values of

$$T_{S}=\max_{1\leq k\leq m-1}\frac{1}{k}\sum_{j=1}^{k}\frac{m{\hat{\varphi }}_{j}^{2}}{\hat{S}\left(0\right)}$$

where $\hat{S}\left(0\right)=\hat{\gamma }\left(0\right)+2\sum_{k=1}^{m-1}\hat{\gamma }\left(k\right)$ is the estimated truncated spectrum at 0. The sample spectrum is the Fourier cosine transform of the estimate of the autocovariance function. The error covariance function at lag *k* is

$$\hat{\gamma }\left(k\right)=\frac{1}{m}\sum_{u=1}^{m-k}\left({\hat{\epsilon }}_{u}-\bar{\hat{\epsilon }}\right)\left({\hat{\epsilon }}_{u+k}-\bar{\hat{\epsilon }}\right)$$

and

$$\hat{\gamma }\left(0\right)=\frac{1}{m}\sum_{u=1}^{m}{\left({\hat{\epsilon }}_{u}-\bar{\hat{\epsilon }}\right)}^{2}$$

where $\hat{\epsilon }$ is the residual from the Fourier estimation in (4).

The p-values of test statistics *Ts* can be calculated from the asymptotic distribution. Since each $m{\hat{\varphi }}_{j}^{2}/\hat{S}\left(0\right)$ has an approximate chi-squared distribution ([33,34]),

$$P\left(T_{S}\leq C\right)\approx \exp\;\left\{-\sum_{j=1}^{\infty }\frac{P\left(\chi_{j}^{2}>\text{jC}\right)}{j}\right\}$$

where $\chi_{j}^{2}$ is a chi-squared random variable with *j* degrees of freedom.

### Clustering differentially expressed genes

All genes for which the null hypothesis of no change has been rejected will undergo clustering analysis, and this will operate on the Fourier domain representation of each expression profile. The sample Fourier coefficient ${\hat{\varphi }}_{j}$ is a weighted average of the observations with

$$\text{Var}\left({\hat{\varphi }}_{j}\right)=O\left(\frac{1}{m}\right)\;\text{and}\;\text{Cov}\left({\hat{\varphi }}_{j},{\hat{\varphi }}_{v}\right)=O\left(\frac{1}{m}\right).$$

By the Central Limit Theorem for dependent data [35], the sample Fourier coefficient ${\hat{\varphi }}_{j}$ is asymptotically normally distributed [36] as *m* → *∞*. With this asymptotic property, we can use the Gaussian mixture model for clustering.

With the large number of genes monitored in these studies, clustering is a key task for microarray data analysis. It seeks to identify groups of genes with similar expression profiles across samples. Clustering can reduce the effort of studying individual genes and more importantly it can unmask the functional groups among genes.

Considering that the empirical Fourier coefficients of the gene profiles have an asymptotic multivariate normal distribution enables the use of an efficient algorithm to compute the posterior probability that a gene belongs to a certain cluster.

The geometric features (shape, volume, orientation) of each group *k* are determined by the covariance matrix *∑*_*k*_ of the Fourier coefficients. A general framework for exploiting the representation of the covariance matrix is done in terms of its eigenvalue composition [37]. Each elliptical model for the covariance matrix is implemented in Mclust [19]. Model-based hierarchical agglomerative clustering is an approach to compute an approximate maximum of the classification likelihood. Each component is weighted by the probability that a sample Fourier coefficient belongs to that component. Our clustering strategy involves model-based agglomerative hierarchical clustering and selection of the model and the number of clusters using approximate Bayes factors with the BIC approximation.

### Performance metrics

For evaluating the performance of clustering algorithms, the adjusted Rand Index [38] and the Silhouette index [39] are used for the simulated data and for the yeast data.

Suppose *T* is the true clustering of a gene expression data set based on domain knowledge and *C* a clustering result given by some clustering algorithm applied to the observed data. Let *a*, *b*, *c* and *d* respectively denote the number of gene pairs belonging to the same cluster in both *T* and *C*, the number of pairs belonging to the same cluster in *T* but to different clusters in *C*, the number of pairs belonging to different clusters in *T* but to the same cluster in *C* and the number of pairs belonging to different clusters in both *T* and *C*. The adjusted Rand index *ARI(T,C)* is defined as.

$$\text{ARI}\left(T,C\right)=\frac{2\left(\text{ad}-\text{bd}\right)}{\left(a+b\right)\left(b+d\right)+\left(a+c\right)\left(c+d\right)}$$

The silhouette width [39] for *i*th sample in the *j*th cluster is defined as.

$$s\left(i\right)=\frac{b\left(i\right)-a\left(i\right)}{\max\left\{a\left(i\right),b\left(i\right)\right\}}$$

where *a* (*i*) is the average distance between the *i*th sample and all other samples included in the *j*th cluster, *b*(*i*) is the minimum average distance between the *i*th sample and all of the samples clustered in *k*th cluster for *k ≠ j*

## Competing interests

The authors declare that there are no competing interests.

## Authors’ contributions

JK initiated this research, outlined the general idea and did the numerical work. RTO improved the method and the simulation scheme. HK accomplished the gene ontology and provided the biological interpretation of the yeast cell cycle data. All authors read and approved the final manuscript.
